# Case Study on the Microbiological Quality, Chemical and Sensorial Profiles of Different Dairy Creams and Ricotta Cheese during Shelf-Life

**DOI:** 10.3390/foods10112722

**Published:** 2021-11-07

**Authors:** Paolo Bellassi, Gabriele Rocchetti, Gianluca Maldarizzi, Gian Paolo Braceschi, Lorenzo Morelli, Luigi Lucini, Fabrizio Cappa

**Affiliations:** 1 Department for Sustainable Food Process, Università Cattolica del Sacro Cuore, Via Emilia Parmense 84, 29122 Piacenza, Italy; paolo.bellassi@unicatt.it (P.B.); gianluca.maldarizzi@libero.it (G.M.); gianpaolo.braceschi@assaggiatori.com (G.P.B.); lorenzo.morelli@unicatt.it (L.M.); luigi.lucini@unicatt.it (L.L.); fabrizio.cappa@unicatt.it (F.C.); 2 Centro Studi Assaggiatori Società Cooperativa, Galleria V. Veneto, 9, 25128 Brescia, Italy

**Keywords:** ricotta cheese, untargeted metabolomics, UHPLC-QTOF-MS, lipids, multivariate statistics

## Abstract

This work investigated the microbiological quality and chemical profiles of two different dairy creams obtained by centrifugation vs. natural creaming separation systems. To this aim, an untargeted metabolomics approach based on UHPLC-QTOF mass spectrometry was used in combination with multivariate statistical tools to find potential marker compounds of the two different types of two dairy creams. Thereafter, we evaluated the chemical, microbiological and sensorial changes of a ricotta cheese made with a 30% milk cream (i.e., made by combining dairy creams from centrifugation and natural creaming separation) during its shelf-life period (12 days). Overall, microbiological analysis revealed no significant differences between the two types of dairy creams. On the contrary, the trend observed in the growth of degradative bacteria in ricotta during shelf-life was significant. Metabolomics revealed that triacylglycerols and phospholipids showed significant strong down-accumulation trends when comparing samples from the centrifugation and natural creaming separation methods. Additionally, 2,3-Pentanedione was among the best discriminant compounds characterising the shelf-life period of ricotta cheese (VIP score = 1.02), mainly related to sensorial descriptors, such as buttery and cheesy. Multivariate statistics showed a clear impact of the shelf-life period on the ricotta cheese, revealing 139 potential marker compounds (mainly included in amino acids and lipids). Therefore, the approach used showed the potential of a combined metabolomic, microbiological and sensory approach to discriminate ricotta cheese during the shelf-life period.

## 1. Introduction

Ricotta belongs to the group of whey dairy products obtained by the coagulation of whey proteins by heat with or without the addition of lactic or citric acids, or calcium and/or magnesium salts to modify the ionic strength [1,2]. It can be considered a typical Italian dairy product, although some variants are produced in other countries, because of the reutilisation of whey from the cheesemaking process [3,4,5,6]. In this regard, the denomination of two products, namely “Ricotta Romana” and “Ricotta di Bufala Campana” is protected by the PDO trademark. Overall, ricotta can be produced by using whey and other milk ingredients from cow, ewe, water buffalo or goat milk, and/or their blends as well. It is usually a fresh product, but it could be ripened or smoked, thus allowing many products to be found on the market. Fresh ricotta is characterised by a high content of water, with a sweet taste of milk and cream and a granular but non-sandy texture, whilst the colour is white, usually depending on the animal species of origin of the raw materials. Lastly, the origin of whey, together with the amount of milk and cream added, influence the protein content of the blend, as well as the addition of whey powders and/or milk proteins [7,8].

Regarding the technological aspects for ricotta production, one important point is represented by the addition of cream to improve the sensorial properties of the product [9]. Therefore, the cream separation represents a very important unit operation in the dairy industry for standardisation purposes [10]. The milk cream is obtained by separating the fat phase of the milk by natural creaming or by centrifugation. Natural creaming takes place in a limited layer overnight at 8–20 °C. It is a natural process occurring for the spontaneous aggregation of milk fat globules. Moreover, natural creaming is a traditional way to reduce the spore-forming bacteria in milk. The spores interact and are entrapped in fat globules during natural creaming. The second way to obtain cream, by the centrifugal separation of fat, can take place both by centrifugation of milk and whey. This process exploits the different density of fat globules compared to the other components of milk. In the centrifugation process, the separation of the fat is almost instantaneous and allows a more concentrated cream that can be used in the production of ricotta to be obtained.

The production process of ricotta cheese includes the following four key points from which all the product variants derive: preparation of raw materials, thermal denaturation and aggregation of proteins, separation of ricotta from the scotta, cooling and packaging. The main raw material used to produce ricotta is given by whey, which is added to a variable amount (5–30%) of milk cream. In addition, 0.5–1.5% of NaCl could be added to improve the whey proteins aggregation. Ricotta is the result of a thermal coagulation process at 80–90 °C followed by the addition of lactic acid or citric acid (1.5–2.5%), depending on the initial acidity of whey. Whey proteins, albumin and globulin, are extremely sensitive to the phenomenon of thermal denaturation. As a result of the process of protein denaturation and consequent aggregation, a curd of modest consistency is formed that incorporates the fat. After 5–20 min, the surfacing curd is collected in draining moulds and transferred to the cold room to rest before the final hot packaging.

The production of ricotta cheese prescribes heating the milk to 85–90 °C, thus inactivating the natural microflora [11]; therefore, it is considered a safe product. However, in case of post-process contamination, the physical–chemical characteristics of ricotta cheese (e.g., high moisture, low salt content and pH values close to neutrality), make the product a suitable substrate for the growth of several harmful microorganisms including pathogenic Enterobacteriaceae (such as *Salmonella* spp.) [12]. It is also important to highlight that the ricotta-making steps and storage conditions could affect the characteristics and oxidative stability of this product, thus influencing the formation of protein- or lipid-oxidation products [13]. Overall, the lipid oxidation process contributes to the potential lowering of the nutritional and sensory properties during the shelf-life of the product, thus leading to the formation of different typical compounds, such as aldehydes, ketones, hydrocarbons, alcohols and acids. In this regard, the content of malondialdehyde (MDA) is often used as a marker of oxidative damage in cheese, as resulting from both the processing and shelf-life conditions [14].

Some previous works evaluated both ricotta quality and yield, as affected by the milk fat content and coagulant type [15]; ricotta cheese from sheep milk fed with different diets [16]; chemical–sensory and volatile profile of ricotta forte cheese [17]; effect of goat breed on the quality characteristics of ricotta cheese [9]; impact of adding probiotic strains on quality characteristics of goat ricotta [18]. However, to the best of our knowledge, limited works have explored the chemical perturbations occurring in ricotta cheese during its shelf-life period (12 days), evaluating also the metabolomic and microbial differences between two different milk creams (i.e., from centrifugation vs. natural creaming separation) added to the product. Therefore, this work was designed to provide new insights into the utilisation of untargeted metabolomics to investigate some variables able to affect the final quality of the ricotta cheese product, as well as to track the chemical changes during the shelf-life of such a perishable product, which can be further used to avoid food safety issues. Moreover, the results could be of practical importance for the producers in terms of deciding which kind of dairy cream to use in the production of ricotta cheese. Finally, the potential correlations existing between the sensory and chemical profiles of this dairy product were inspected.

## 2. Materials and Methods

### 2.1. Milk Cream and Ricotta Samples

In this work, we analysed a total of 6 dairy creams divided into both natural creaming-derived (from the manufacturing of hard-cheese) and centrifuge-derived (from residual milk whey) creams intended to produce ricotta, and 4 ricotta samples produced with mixtures of the above-mentioned creams (50% derived from natural creaming and 50% derived by centrifugation). Both creams and ricotta samples were collected in different batches over a period of 3 weeks to increase biological variability. In particular, the following acronyms were used: B1-CA, B2-CA and B3-CA (natural creaming-derived creams); B1-CC, B2-CC and B3-CC (centrifugation-derived creams). Additionally, the natural creaming-derived creams were obtained following a long-ripening hard cheese production process (where cream is separated from milk and then hard cheese is produced from semi-fat milk), whilst the centrifuged creams were obtained by centrifuging the whey remaining after cheese manufacturing. The samples considered belonged to those usually arriving for routine analysis at our laboratory at the Università Cattolica del Sacro Cuore (Piacenza, Italy). The phases relating to sampling involved the collection of different creams using special containers (100 mL), while the ricotta samples were collected on the day after processing. Additionally, the following acronyms (corresponding to the different batches) were used for the ricotta samples under investigation: B1-R, B2-R, B3-R and B4-R. Finally, the different ricotta batches were analysed just after being packaged (t0), after 6 days (t6) and 12 days (t12) of shelf-life at a temperature of 4 °C. The ricotta samples analysed were composed of 30% (*w*/*w*) of milk cream, made up 50% of cream from natural creaming and 50% of cream from centrifugation.

### 2.2. Bacterial Counts

Ricotta and cream samples were microbiologically characterised by counting different classes of microorganisms. A 25-g sample was diluted with 225 mL of peptone salt solution consisting of 9 g/L NaCl and 1 g/L peptone; then, the 10-fold dilution method was used. Samples were analysed for total aerobic mesophilic microorganisms (Total Mesophilic Count, TMC) using Milk Plate Count Agar (MPCA; Thermo Fisher Scientific™, Oxoid™, Waltham, MA, USA), a non-selective and non-differential medium. Seeding was by inclusion, and incubation was at 30 °C for 48 h. For citrate-fermenting microorganisms (CFM), the medium used for counting was de Man, Rogosa and Sharpe agar (MRS Agar; BD™, Difco™, Franklin Lakes, NJ, USA) with 15% calcium citrate. Calcium citrate is the salt of citric acid, which occurs as an odourless white powder. Plates were made according to the following protocol: 7.5 g of calcium citrate was suspended in 50 mL of water using ultrasound for 5 min to obtain a stable suspension. To eliminate coarse grains, the suspension was filtered through a paper filter (Whatman^®^, Cytiva, Marlborough, MA, USA). Subsequently, the suspension was autoclaved at 121 °C for 15 min and then mixed with MRS agar autoclaved at 121 °C for 15 min. Subsequently, the suspension was autoclaved and then mixed with MRS agar autoclaved at 121 °C for 15 min at a temperature of 55°C. Seeding was performed by inclusion and incubation was performed at 30 °C for 48 h under anaerobic conditions. The medium used for counting Enterobacteriaceae (ENTERO) was the Violet Red Bile Glucose Agar (VRBGA; Thermo Fisher Scientific™, Oxoid™, Waltham, MA, USA). This medium was heated to boiling point without being autoclaved. Seeding was by inclusion and incubation was at 37 °C for 24 h. For the enumeration of *Pseudomonas* spp. (PSEU), a selective medium *Pseudomonas* agar base (PAB; Thermo Fisher Scientific™, Oxoid™, Waltham, MA, USA) with 5 mL of glycerol autoclaved at 121 °C for 15 min was used. Just prior to the fabrication of the Petri dishes, CFC Supplement (Thermo Fisher Scientific™, Oxoid™, Waltham, MA, USA) was added to the medium. Incubation was carried out at a temperature of 30 °C for 48 h.

### 2.3. Extraction Process for Untargeted Metabolomics Analysis

The extraction of metabolites from both milk creams and ricotta samples was carried out as previously reported [19,20], with some modifications. Briefly, 2 g of each sample was extracted using a 10-millilitre mixture containing 80:20 (*v*/*v*) methanol:water, added with 0.1% of formic acid, with an Ultra-Turrax homogeniser (IKA-Werke, Staufen im Breisgau, Germany) for 4 min at room temperature. Next, the samples were centrifuged at 12,000× *g* for 10 min at 4 °C and the supernatants were incubated overnight in a freezer (−18 °C) following the addition of a 5% TCA solution, to remove large biomolecules (such as proteins). The supernatants were then filtered through 0.2-micrometre cellulose membranes and transferred to amber vials for the further metabolomic analysis. Each sample was analysed considering three biological replications.

### 2.4. Untargeted UHPLC-QTOF-MS Analysis

In this work, high-resolution mass spectrometry analysis was performed on a hybrid quadrupole-time-of-flight instrument (Agilent 6550 iFunnel), coupled to an ultra-high-pressure liquid chromatographic system (Agilent 1200 series) equipped with a binary pump and a JetStream electrospray source to investigate the metabolomic profile in both milk cream and ricotta samples. The mass spectrometer worked in positive polarity and SCAN mode (range 80–1200 *m*/*z*), with nominal resolution at 40,000 FWHM. Chromatographic separation was conducted under a water–methanol gradient elution (6 to 94% methanol in 32 min) on an Agilent Zorbax Eclipse Plus C18 column (50 × 2.1 mm 1.8 μm). The QTOF conditions were optimised in previous published papers [19,20]. The sequence was randomised, injecting 6 μL for each sample replication and blank samples (consisting in extraction solvent only). The raw data were processed using the software Mass Hunter Profinder B.06 (from Agilent Technologies, Santa Clara, CA, USA), exploiting the comprehensive “Milk Composition Database” [21] and working based on a find-by-formula algorithm. Overall, mass features’ annotation was based on an accurate mass and isotope pattern (i.e., exact masses, relative abundances and *m*/*z* spacing). A filter by frequency data reduction was applied, thus retaining features in at least 75% of replications within a treatment. In our analytical conditions and according to COSMOS Metabolomics Standards Initiative, a confidence Level 2 in identification (i.e., putatively annotated compounds) was achieved [22]. Finally, according to Foroutan et al. [21], the term “metabolite species” was used for those molecules with non-unique chemical formulas or masses (e.g., lipid derivatives), while “unique compound structures” were those compounds with a unique chemical formula or mass.

### 2.5. Sensory Analysis of the Ricotta Cheese Samples

In this work, sensory testing was performed on one batch of ricotta samples produced by mixing the creams obtained by both spontaneous separation and centrifugation, differing in the shelf-life period as well (i.e., 0, 4, 7 and 9 days). In particular, the analysis was conducted in the sensory laboratory of the Università Cattolica del Sacro Cuore (Piacenza, Italy), considering a thirty-member (voluntary) panel (15 male and 15 female 20–30-year-olds). The panellists were staff and graduate students from our department. The judges worked completely blindfolded; duplicate samples were offered in a random order, labelled with random five-digit codes. The sensory evaluations were carried out using the Big Sensory Test (BST) method, according to the characteristic phases of the sensory analysis, starting from the visual analysis, then olfactory, tactile taste and the evaluation of the retro-olfactory perceptions. This method first enables a descriptive profile of the products to be identified and then qualitative attributes to be determined. Finally, the validation of the results was conducted considering three different indicators, namely, (a) control of the reliability of the descriptors (cut-off > 6); (b) control of the effectiveness of the judges (in terms of both reliability and quality); control of the sensory form (based on a 9-point hedonic scale) used to measure the sensory descriptors.

### 2.6. Statistical Analysis

Microbiological data of cream samples were expressed as mean and standard deviation of analytical triplicate (n = 3) and processed through “t student” analysis comparing natural creaming-derived creams vs. centrifuge-derived batch averages. The results were expressed as a mean and standard deviation of analytical triplicate (n = 3) and were processed using analysis of variance (ANOVA) and Tukey Test HSD, using IBM SPSS Statistics 26.0 software (IBM, Armonk, NY, USA). The metabolomics dataset containing the annotated compounds was elaborated using the software Mass Profiler Professional B.12.06 (from Agilent Technologies, Santa Clara, CA, USA) as previously described [23]. The annotated compounds were filtered by abundance (retaining those compounds with an area > 5000), Log2 transformed, normalised at 75th percentile and then each abundance baselined against its median in all samples. Thereafter, several multivariate statistical approaches were used, namely, unsupervised principal component analysis (PCA), hierarchical clustering and orthogonal projection to latent structures discriminant analysis (OPLS-DA). In particular, the unsupervised PCA and clustering analyses were performed using the Mass Profiler Professional B.12.06 software, whilst the OPLS-DA and the following selection of the discriminant variables (VIP) were performed using the SIMCA 13 software (Umetrics, Malmo, Sweden). For the latter, the predictive and orthogonal components of the variation between groups were separated, and then the presence of significant outliers in the prediction model was investigated by Hotelling’s T-squared distribution. The model validation parameters (i.e., goodness-of-fit and goodness-of-prediction) were also recorded. The prediction model was cross-validated using a cross-validation ANOVA (*p* < 0.01), whereas a permutation plot was produced to exclude model overfitting (number of random permutations = 100). Finally, the VIP selection method was performed using a VIP score > 1 as a cut-off for prediction. Moreover, each discriminant compound was provided together with its Log2FC value resulting from the fold-change (FC) analyses.

## 3. Results and Discussion

### 3.1. Microbiological Analyses

The microbiological characterisation was carried out on three batches of cream samples produced using the natural creaming technique and three batches of cream produced using the centrifugation process. The results are reported in Table 1 and Table 2. The TMC values in the cream samples showed a range between 6 and 8 log cfu/g. Additionally, the average Enterobacteriaceae (ENTERO) contamination levels of the various centrifuge and cream samples ranged from 5 to 7 log CFU/mL. Regarding the CFM count, the average charge ranged from 4 to 6 log CFU/mL. The contaminating microorganisms belonging to the genus *Pseudomonas* had levels ranging from 5 to 7 log CFU/mL. No significant differences were found between the batches obtained with the natural creaming technique compared to those obtained with the centrifuge technique. The microbiological analyses were also performed on the corresponding ricotta samples obtained by adding 30% of a 1:1 (*w*/*w*) mixture of cream obtained by the two considered creaming processes. Data were collected immediately at the time of packaging, then at mid shelf-life (6 days) and at the end of the shelf-life period (12 days). At the time of packaging (T0), there was a constant presence of total bacterial count, with values around 2 log CFU/g. The bacterial counts in the *Enterobacteriaceae* and *Pseudomonaceae* families were below 10 and below 100 CFU/g, respectively. At the half shelf life (T6) conducted under refrigerated conditions (T = 4 °C), the values for the total bacterial count showed a significant (*p* < 0.05) two logarithms increase compared with the data obtained in the previous analysis (i.e., start of shelf-life). Regarding the count of microorganisms belonging to the *Enterobacteriaceae* and *Pseudomonaceae* families, some contaminations were found that may potentially affect the stability of the product. At the end of shelf-life period, it showed a significant (*p* < 0.05) increase in the total microbiological load with levels above 7 log CFU/g. It also showed contamination of the different ricotta cheese samples exceeding 4 log CFU/g for bacteria of the *Enterobacteriaceae* and *Pseudomonaceae* family.

The values of TMC and *Pseudomonaceae* measured during the shelf-life period followed similar trends to those already reported by Sattin et al. [24]. It can be hypothesised that the initial bacterial contamination is strongly influenced by the microbiological quality of the creams used in the ricotta production process. The microbiological overview of this sample batch showed that both cream and ricotta cheese at the end of shelf life were characterised by higher levels of TMC, *Pseudomonaceae* and *Enterobacteriaceae*. The detected levels are in accordance with other research studies conducted on this type of product. Additionally, Scatassa et al. [4] evaluated Italian ricotta samples during a long period of 15 years of production. The values we found are above the average reported by these authors. Additionally, we noted that values around 2 log TMC and <10 for *Pseudomonaceae* and *Enterobacteriaceae* at the beginning of the shelf-life increase during storage at 4 °C. This evidence suggests the inefficiency of low temperatures for the microorganisms’ growth inhibition. Indeed, some authors investigated alternative solutions to mitigate this phenomenon, e.g., Ricciardi et al. [25] proposed the utilisation of UV light sources to improve the shelf-life of ricotta cheese.

### 3.2. Metabolomic Discrimination between Creams Obtained by Natural Creaming and Centrifugation

**Figure 1:**
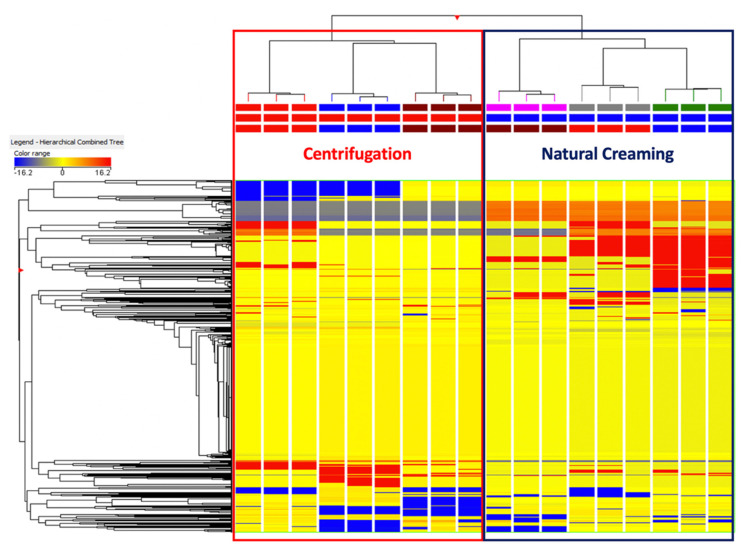
Unsupervised hierarchical cluster analysis (HCA) based on fold-change heat map (similarity: Euclidean; linkage rule: Ward) for the different milk creams resulting from centrifugation and natural creaming.

In this work, untargeted metabolomics based on UHPLC-QTOF mass spectrometry was firstly used to detect the differences and similarities in the metabolomic profile of the creams obtained using natural creaming and centrifugation processes. To reduce the complexity of the metabolomic dataset [26], unsupervised multivariate statistical approaches, namely, hierarchical clustering analysis (HCA) and principal component analysis (PCA), were firstly used. The heat map produced from the fold-change (FC) distribution of each milk cream metabolites is reported as Figure 1.

**Figure 2:**
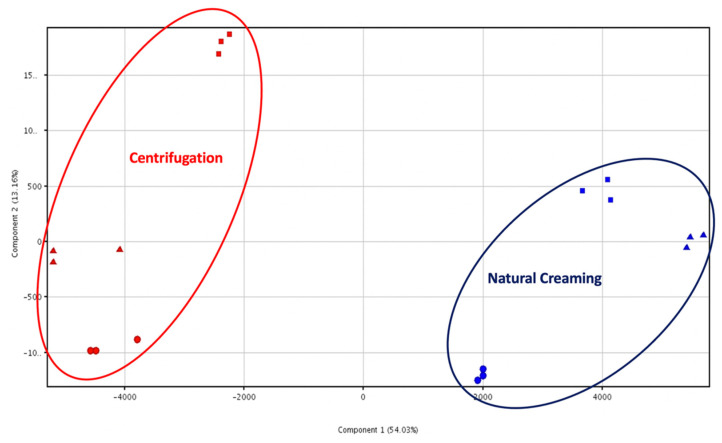
Unsupervised principal component analysis (PCA) considering the different separation technique (i.e., centrifugation vs. natural creaming). The individual sample replications (n = 3) are provided into the PCA score plot.

Interestingly, the heat map (Figure 1) revealed the great hierarchical importance of the separation technique, outlining clear differences in the metabolomic profile of milk creams from centrifugation and natural creaming. This was particularly evident when considering a specific class of metabolites that clearly discriminated (in terms of abundances) the natural creaming separation. Moreover, we also detected a secondary clustering tendency between the creams belonging to the same group, being some cluster of metabolites represented in only one sample (Figure 1). Therefore, two important pieces of information were extrapolated using the HCA heat map, namely, (a) a clear difference imposed using the separation technique on the metabolomic profile of milk creams, and (b) a certain degree of variability in the creams belonging to the same group. As the next evaluation, a PCA was used to minimise this variability on two principal components. As can be observed from Figure 2, the unsupervised PCA score plot explained more than 67% of the cumulative variability with two principal components, thus confirming the marked differences in the chemical profile of both the dairy creams under investigation.

**Figure 3:**
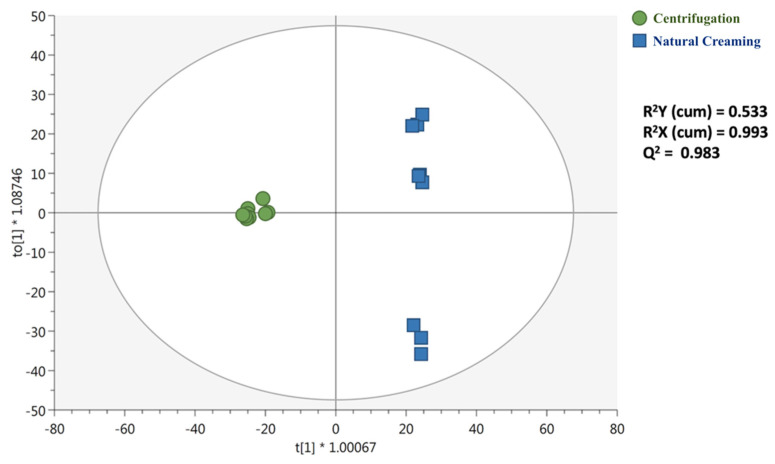
Orthogonal projections to latent structures discriminant analysis (OPLS-DA) score plot considering the different separation technique (i.e., centrifugation vs. natural creaming). The individual sample replications (n = 3) are provided in the OPLS-DA score plot.

Afterwards, to better investigate the contribution of each group of metabolites for discrimination purposes, the multivariate supervised orthogonal projection to latent structures discriminant analysis (OPLS-DA) was carried out. This statistical tool is particularly able to remove the variation not directly correlated with Y in an X matrix (orthogonal signal correction), thus considering only the Y-predictive variation. The OPLS-DA score plot is provided as Figure 3.

Interestingly, we noticed that the orthogonal latent vector clearly discriminated milk cream samples from the two separation methods; however, a higher degree of variability could be highlighted for the natural creaming group, thus allowing us to postulate that a spontaneous separation is a technological process carrying the most variability. Taken together, both unsupervised and supervised multivariate statistical approaches allowed us to discriminate milk creams according to the technological process chosen. In our experimental conditions, the OPLS-DA model cross-validation and goodness parameters were excellent, recording 0.933 for the goodness-of-fit (R^2^Y) and 0.983 for the goodness-of-prediction (Q^2^), with a significant cross-validation ANOVA *p*-value (2.47 × 10^−^^11^) and permutation plot (Table S1). Therefore, the absence of over fitting together with high correlation and prediction abilities confirmed the potential and robustness of the model based on milk cream metabolites, for investigating the main differences as related to the technological separation process.

Afterwards, the discriminant metabolites imposed by the separation process (i.e., centrifugation vs. natural creaming) were selected using the VIP approach. This latter provides the VIP compounds, i.e., the best marker compounds of the prediction model built, characterised by a VIP discriminant score > 1. Overall, 541 compounds (including several isomeric forms of lipids) possessed a VIP score > 1. These compounds were reduced to 72 when excluding the isomeric forms of lipids and other metabolites and considering a VIP score > 1.2 (Table 3).

In particular, the highest VIP scores were found for isomeric forms of triacylglycerols, such as TG(15:0/24:1(15Z)/18:1(9Z)), TG(14:0/18:3(9Z,12Z,15Z)/19:0)[iso6], TG(13:0/18:0/20:2(11Z,14Z))[iso6] and TG(13:0/18:2(9Z,12Z)/21:0)[iso6]. Overall, a summarising table reporting all the discriminant VIP compounds can be found in the Supplementary Material (Table S1). Interestingly, those triacylglycerols and phospholipids possessing the highest discrimination potential showed strong down-accumulation trends when comparing samples from the centrifugation and natural creaming separation methods (Table S1). Regarding marker compounds specifically characterising each separation method, we found that 4-Hydroxyphenyl-beta-glucopyranoside (a phenolic glycoside), 10Z-Pentadecenoic acid (belonging to fatty acyls) and 3-Sulfinoalanine (belonging to the class of L-alpha-amino acids) exclusively characterised the centrifugation process, with averaged LogFC values of 20.8, 20.4 and 19.3, respectively. Looking at specific marker compounds of the natural creaming method, we found that LysoPC(16:0) and isomeric forms of triacylglycerols were particularly retained following the spontaneous separation. Therefore, as expected, the VIP markers of the OPLS-DA model revealed a higher discriminant weight and a wider distribution of different lipid classes (such as fatty acyls and triradylglycerols). Moreover, it is well known that milk whey (used to produce centrifuge creams) is mainly characterised by lactose, proteins (such as lactalbumin), minerals and lipids (the latter in trace amounts) [27,28]. Regarding the discriminant metabolites observed in natural creaming-derived cream samples, the largest variability observed from multivariate statistics demonstrated the impossibility to standardise the separation conditions. These cream samples have been obtained considering the milk of the evening, stored until the morning in large tanks, where the natural creaming of the lipidic part takes place. Therefore, the discriminant weight of lipids and derivatives outlined for natural creaming-derived cream samples could lead to a major contribution to the final aroma of the ricotta samples when compared to centrifuge creams, thus supporting the need to mix these by-products to maximise the sensorial attributes of the final product.

### 3.3. Changes of Chemical Composition of Ricotta during Shelf-Life

**Figure 4:**
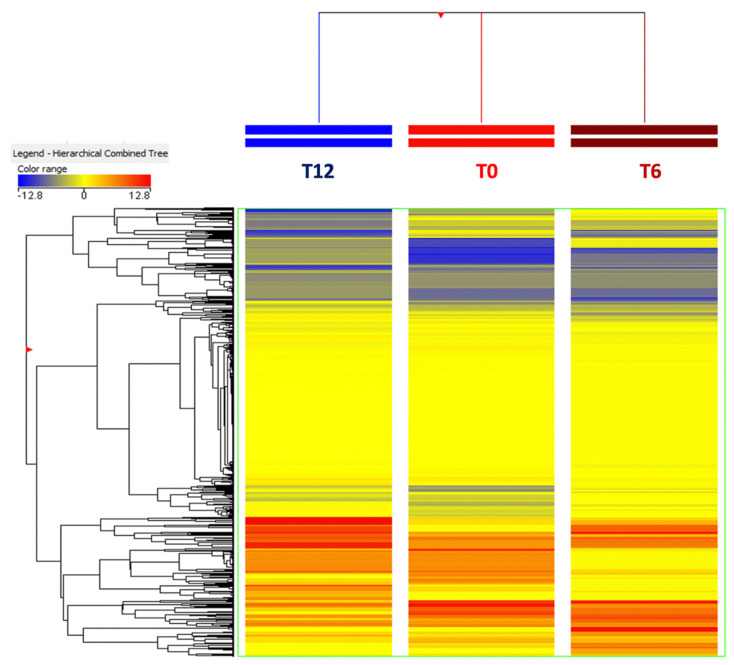
Average unsupervised hierarchical cluster analysis (HCA) built considering the shelf-life period of the ricotta samples (T0 = 0 days; T6 = 6 days; T12 = 12 days).

As the next evaluation, the metabolomics approach was used to explore the changes of the main chemical classes in ricotta cheese during 12 days of shelf-life period. Those creams (both from centrifugation and natural creaming) resulting from the same sampling day were used to formulate three ricotta samples that were monitored overtime, while a fresh ricotta sample was used as a control for the following sensory analysis. Overall, an averaged unsupervised clustering was used to inspect the changes of ricotta compounds during the shelf-life period. As can be observed from the heat map in Figure 4, a clear impact of the shelf-life period could be outlined (as evident from the average fold-change variations represented by the blue and red colours).

**Figure 5:**
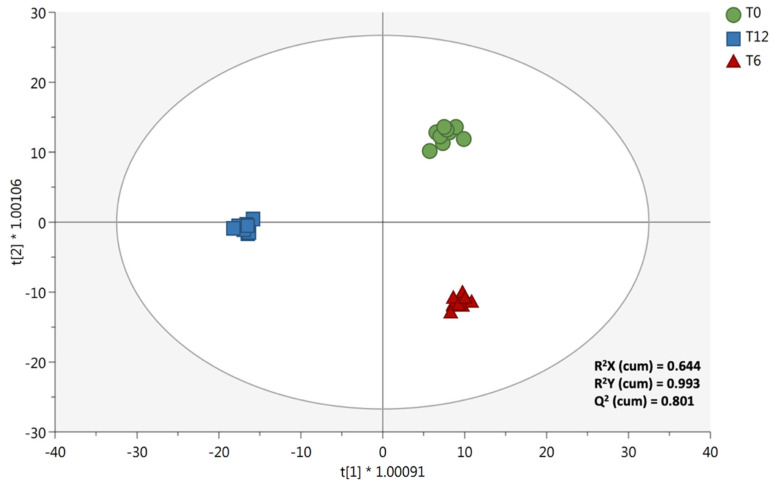
Supervised OPLS-DA score plot built considering the shelf-life period of the ricotta samples as class discrimination parameter.

Therefore, to extrapolate the changes of ricotta chemical profile during the shelf-life period, a following supervised statistical approach was used. Overall, ricotta samples were analysed just after being packaged (t0), after 6 days (t6) and 12 days (t12) of shelf life at a temperature of 4 °C, by adding a 30% milk cream, obtained by mixing 50% of the cream from the natural creaming and 50% of cream from the centrifugation processes. The OPLS-DA score plot built considering the three ricotta samples formulated with the addition of the different milk creams is represented in Figure 5.

Overall, the class membership criterion used to build the prediction model was the shelf-life period. It was clear from the OPLS-DA score plot that ricotta samples possessed distinct chemical profile at each shelf-life time-point considered. The model was again found to possess more than acceptable goodness parameters, with the goodness of fit = 0.993 and the goodness of prediction = 0.801. Additionally, the model possessed a cross-validated *p* value < 0.05, while permutation testing (number of random permutations = 100) and Hotelling’s T^2^ distribution allowed the exclusion of both overfitting and strong outliers, respectively. The variation of the main chemical classes annotated in the ricotta samples during the shelf-life period (12 days) was then evaluated by inspecting the Log2FC values of the discriminant compounds, as reported in Table 4.

As the first consideration, the VIP selection method following the supervised OPLS-DA allowed 139 discriminant compounds (excluding the isomeric structures), that are reported in supplementary material (Table S1), to be detected, together with their MS spectra. Overall, the group composed by amino acids, peptides and analogues consisted of 25 compounds, with the peptide Val-Pro-Pro showing the highest discrimination potential (VIP score = 1.89) and a negative variation at 12 days of shelf-life (Log2FC = −0.29). Additionally, the compound Prolyl-Histidine showed the highest increase during the entire shelf-life, recording Log2FC values of 4.37 (at 6 days) and 16.71 (at 12 days). Instead, an opposite trend (i.e., decrease in both time points considered) was outlined for glutamic acid, recording Log2FC values of −3.82 (at 6 days) and −9.05 (at 12 days).

Regarding other chemical classes and compounds, benzenoids showed an average down-accumulation at 12 days of shelf life (Log2FC = −1.44), with cresol (i.e., a methylated phenol) possessing the highest discrimination potential (VIP score = 1.51), whilst the phenolic glycoside 4-Hydroxyphenyl-beta-glucopyranoside (classified among the carbohydrates and carbohydrate conjugates) showed a strong up-accumulation at the end of shelf-life period (Log2FC = 11.82). Interestingly, the group of polyphenols annotated using HRMS showed an average down-accumulation during the shelf-life period; however, the compounds showing the highest VIP score (Table 4), namely, trans-cinnamic acid (a phenolic acid), showed an increase moving from 6 up to 12 days of shelf-life, recording Log2FC values of 2.21 and 3.33, respectively. According to our findings, the most represented class of discriminant compounds during shelf-life consisted of lipids and derivatives (Table 4 and Table S1). We found several discriminant classes, namely, fatty acyls, diacylglycerols, glycerophosphocholines and triradylglycerols (Table 4). As reported in Table 4, at 12 days of shelf-life, glycerophosphocolines showed an average down-accumulation trend (Log2FC = −2.31), while fatty acyls, diacylglycerols and triradylglycerols were all characterised by average increasing trends, with the maximum Log2FC value recorded for diacylglycerols (i.e., 4.42). 

Looking at other compounds, the group composed by purines, pyridines and pyrimidines showed few variations, recording an average down-accumulation (i.e., −0.52) at 12 days of shelf-life. Finally, we found 2,3-Pentanedione (also known as acetylpropionyl) among the discriminant compounds characterising the shelf-life period (Table S1) being characterised by a VIP score = 1.02. This compound is a diketone, widely described in the literature as related to sensorial descriptors, such as buttery, cheesy, sweet, nutty, fruity, creamy and caramel. In our experimental conditions, it was characterised by significant up-accumulation trends at both 6 days (Log2FC = 4.69) and 12 days (Log2FC = 4.61). Ketones mainly derive from biochemical mechanisms involving the lysis of triglycerides and the oxidation of saturated free fatty acids, with the consequent production of ketoacids that are decarboxylated to ketones that, in turn, can be reduced to obtain alcohols [29].

### 3.4. Sensory Analysis

The sensory data were expressed as medians resulting from the evaluations of all the panellists. The validation of the medians was based on a reliability index for each descriptor and expressed on a scale from 0 to 10, where 0 corresponds to the minimum and 10 to the maximum. According to this approach, the minimum cut-off value to consider a sensorial descriptor as sufficient was six. The data obtained were then processed by comparing the ricotta samples with a different shelf-life period, thus highlighting the most specific and characteristic sensorial descriptors. These latter were mainly paste homogeneity, compactness, olfactory intensity, meltability, milk and paste. Friedman’s ANOVA test was then used to check for statistically significant differences in the group of the ricotta samples analysed, and then the non-parametric LSD was used to check the differences between the samples. Interestingly, only the “compactness” attribute provided a significant difference, revealing an overall decrease during the shelf-life of the product. Regarding other sensory descriptors, the olfactory intensity showed a decrease near the expiry date of the ricotta samples (sample R1 and sample R3). This aspect is also linked to a reduction in the perception of the milk within the product and to the pastry descriptor that is linked to the perception of milk cream and other “vanilla” descriptors.

## 4. Conclusions

This work investigated the untargeted chemical profile of dairy creams obtained using centrifugation vs. natural creaming separation processes. The metabolomics-based approach successfully allowed us to discriminate between the two products, revealing distinctive chemical signatures. In this regard, a higher degree of variability was highlighted for the natural creaming group, thus confirming that a spontaneous separation is a technological process carrying most of the variability when considering the final chemical composition. The most discriminant marker compounds between the two creams were found to be lipids (such as triacylglycerols and phospholipids) and other lower-molecular weight metabolites (such as phenolics and amino acids). Moreover, a strong down-accumulation of lipids in the centrifuged cream samples was outlined. Thereafter, the same creams were used to produce ricotta samples, thus evaluating both the changes of their untargeted chemical profile and sensorial behaviour during a shelf-life period of 12 days. Multivariate statistics (based on both supervised and unsupervised tools) showed a clear impact of the shelf-life period on the changes of some classes of metabolites (i.e., 139 compounds), such as lipids (i.e., fatty acyls, diacylglycerols, glycerophosphocholines and triradylglycerols), peptides and amino acids. The metabolomics-based approach also allowed us to identify some typical sensorial descriptors of ricotta cheese, with 2,3-Pentanedione (also known as acetylpropionyl) being the most discriminant one. The use of culture-dependent microbiological techniques allowed us to also characterise the samples in terms of possible contamination both at an ingredient level (milk cream) and during the ricotta cheese shelf-life, confirming the trends found in other works. Finally, the sensory analysis revealed that only the “compactness” attribute provided significant differences among the panellists, showing an overall decrease during the shelf-life of the product. Therefore, taken together, our preliminary findings demonstrate the suitability of cheese metabolomics to investigate some variables that are able to affect the final quality of the product, in terms of both chemical and sensory attributes.

## Figures and Tables

**Table 1:** Microbiological counts of the natural creaming-derived and centrifuge-derived creams samples. Data are expressed as mean values ± standard deviation (n = 3) of each batch.

Samples	TMC Log10 (cfu/g)	CFM Log10 (cfu/g)	ENTERO Log10 (cfu/g)	PSEU Log10 (cfu/g)
B1-CA	7.27 ± 0.07	5.95 ± 0.04	5.34 ± 0.05	7.11 ± 0.07
B2-CA	6.62 ± 0.15	5.26 ± 0.15	5.93 ± 0.03	5.98 ± 0.20
B3-CA	7.11 ± 0.05	5.80 ± 0.12	6.99 ± 0.07	6.97 ± 0.07
B1-CC	6.82 ± 0.03	4.90 ± 0.06	5.40 ± 0.44	4.30 ± 0.01
B2-CC	5.86 ± 0.23	4.12 ± 0.01	4.25 ± 0.24	5.12 ± 0.01
B3-CC	8.21 ± 0.06	6.85 ± 0.02	6.42 ± 0.09	6.82 ± 0.01
Significance CA vs. CC	ns	ns	ns	ns

B1-CA, B2-CA and B3-CA (natural creaming-derived creams); B1-CC, B2-CC and B3-CC (centrifuge-derived creams); ns = not significant (*p* < 0.05). *Abbreviations*: TMC = total mesophilic count; CFM = citrate-fermenting microorganisms; ENTERO = *Enterobacteriaceae* selective count; PSEU = *Pseudomonas* selective count.

**Table 2:** Microbiological counts of the different ricotta samples during the shelf-life period. Data are expressed as mean values ± standard deviation (n = 3) of each batch.

Samples	TMC Log_10_(cfu/g)	ENTERO Log_10_(cfu/g)	PSEU Log_10_(cfu/g)
T0			
B-1R	1.93 ± 0.08 ^a^	<10 ^a^	<100 ^a^
B-2R	2.54 ± 0.04 ^a^	<10 ^a^	<100 ^a^
B-3R	2.58 ± 0.04 ^a^	<10 ^a^	<100 ^a^
B-4R	2.53 ± 0.05 ^a^	<10 ^a^	<100 ^a^
T6			
B-1R	4.45 ± 0.08 ^b^	<10^a^	3.79 ± 0.27 ^b^
B-2R	5.06 ± 0.02 ^b^	<10^a^	3.11 ± 0.06 ^b^
B-3R	5.06 ± 0.01 ^b^	3.63± 0.06 ^b^	3.00 ± 0.01 ^b^
B-4R	5.89 ± 0.07 ^b^	4.18 ± 0.14 ^b^	3.65 ± 0.02 ^b^
T12			
B-1R	7.17 ± 0.08 ^c^	4.63 ± 0.02 ^c^	6.65 ± 0.13 ^c^
B-2R	7.41 ± 0.04 ^c^	5.63 ± 0.03 ^c^	7.08 ± 0.03 ^c^
B-3R	7.94 ± 0.04 ^c^	6.79 ± 0.04 ^c^	7.65 ± 0.05 ^c^
B-4R	7.64 ± 0.07 ^c^	7.16 ± 0.01 ^c^	7.70 ± 0.02 ^c^
Significance (shelf-life period)	*p* < 0.05	*p* < 0.05	*p* < 0.05

The letters a, b and c indicate significant differences between the batch averages of ricotta cheese samples at different times of shelf-life. *Abbreviations*: TMC = total mesophilic count; ENTERO = *Enterobacteriaceae* selective count; PSEU = *Pseudomonas* selective count.

**Table 3:** VIP discriminant compounds resulting from the OPLS-DA based on the discrimination between creams obtained by natural creaming (NC) vs. centrifugation (C) methods. The VIP score and the Log2FC value are also provided.

Chemical Class	Discriminant Compounds (OPLS-DA)	VIP Score (OPLS-DA)	Log2FC (C) vs. (NC)
Amino acids, peptides, and analogues	Serylmethionine	1.492	−15.92
	3-Sulfinoalanine	1.364	19.35
	Ergothioneine	1.320	8.35
	3-Methylhistidine	1.287	12.25
	Methionine sulfoxide	1.254	2.82
	Val-Pro-Pro	1.237	−0.55
	N-Formyl-L-methionine	1.234	0.44
	Pretyrosine	1.222	−11.91
	L-Homoserine	1.221	2.18
	Phe-Pro-Ile	1.211	−10.79
	Pro-Pro-Phe	1.208	−12.51
	Aspartyl-Valine	1.205	−9.94
	2-Aminoisobutyric acid	1.200	−9.98
Fatty Acyls	Tetracosapentaenoic acid (24:5*n*-6)	1.362	−6.53
	10Z-Pentadecenoic acid	1.323	20.41
	Isobutyrylcarnitine	1.285	0.40
	Butyrylcarnitine	1.284	0.41
	Citraconic acid	1.268	18.60
	Myristic acid	1.220	−11.74
	12-Methyltridecanoic acid	1.213	−11.69
Triradylglycerols	TG(15:0/24:1(15Z)/18:1(9Z))	1.520	−16.00
	TG(14:0/18:3(9Z,12Z,15Z)/19:0)[iso6]	1.513	−12.45
	TG(13:0/18:0/20:2(11Z,14Z))[iso6]	1.503	−11.95
	TG(13:0/18:2(9Z,12Z)/21:0)[iso6]	1.497	−0.97
	TG(13:0/20:1(11Z)/22:5(7Z,10Z,13Z,16Z,19Z))[iso6]	1.494	−16.40
	TG(13:0/18:0/22:4(7Z,10Z,13Z,16Z))[iso6]	1.493	−11.56
	TG(17:0/18:4(6Z,9Z,12Z,15Z)/20:5(5Z,8Z,11Z,14Z,17Z))[iso6]	1.489	−15.76
	TG(16:0/16:1(9Z)/20:0)[iso6]	1.485	−6.68
	TG(14:0/18:0/18:0)	1.481	−8.56
	TG(18:1(11Z)/16:0/18:1(11Z))[iso3]	1.480	−6.47
	TG(14:0/14:1(9Z)/15:0)	1.455	−17.29
	TG(14:1(9Z)/14:1(9Z)/16:1(9Z))	1.441	−12.48
	TG(12:0/16:0/16:1(9Z))[iso6]	1.404	−16.56
	TG(13:0/18:0/22:5(7Z,10Z,13Z,16Z,19Z))[iso6]	1.376	−11.30
	TG(18:0/18:0/18:1(9Z))[iso3]	1.333	−17.10
	TG(18:0/18:0/18:0)	1.272	−16.49
	TG(20:1(11Z)/20:1(11Z)/20:1(11Z))	1.270	−10.12
	TG(12:0/12:0/20:2(11Z,14Z))[iso3]	1.258	−16.49
	TG(14:0/14:1(9Z)/16:1(9Z))[iso6]	1.246	−16.44
	TG(13:0/17:0/18:3(9Z,12Z,15Z))[iso6]	1.226	−11.22
	TG(18:1(9Z)/15:0/o-18:0)	1.224	−11.96
	TG(16:0/18:0/20:4(5Z,8Z,11Z,14Z))[iso6]	1.219	−11.57
	TG(17:0/18:2(9Z,12Z)/18:3(6Z,9Z,12Z))[iso6]	1.206	−5.52
Polyphenols and derivatives	5,7,8,4′-Tetrahydroxyisoflavone	1.362	−7.32
	Homovanillic acid	1.323	9.18
	2-Pyrocatechuic acid	1.290	9.82
	Equol	1.277	0.40
	3′,4′,7-Trihydroxyisoflavanone	1.273	8.07
	2-Methylhippuric acid	1.297	0.44
	4-Hydroxyphenyl-beta-glucopyranoside	1.495	20.77
Sugars and sugars derivatives	Glucose 1-phosphate	1.389	18.16
	Galactose 1-phosphate	1.248	18.33
	Isopropyl beta-D-glucoside	1.512	−7.73
	Maltotetraose	1.402	0.38
	Maltotriose	1.385	11.76
Other lipids and derivatives	Galactosylceramide (d18:1/20:0)	1.454	−16.92
	PC(18:2(9Z,12Z)/20:4(5Z,8Z,11Z,14Z))	1.495	−13.98
	PC(o-16:1(9Z)/18:2(9Z,12Z))	1.389	−6.81
	LysoPC(16:0)	1.278	−12.59
	Cer(d18:0/22:1(13Z))	1.484	−15.54
Other metabolites	Ethyl furoate	1.482	13.33
	(E,E)-2,4-Hexadienal	1.249	19.02
	Tyramine	1.242	4.33
	Phenylacetaldehyde	1.258	16.74
	Hydroxyphenyllactic acid	1.307	3.53
	1-Methyladenosine	1.273	−16.67
	Nicotinic acid	1.221	2.18
	Uridine 5′-monophosphate	1.331	16.06
	Uracil	1.299	17.37
	Pantothenic acid	1.285	0.37
	2b,3a,7a,12a-Tetrahydroxy-5b-cholanoic acid	1.201	−11.93
	Loganin	1.224	−11.24

**Table 4:** Changes (expressed as average Log2 Fold-Change values) during shelf-life of the chemical classes annotated using UHPLC-QTOF mass spectrometry. The most discriminant compound (VIP marker) for each class is also reported.

Chemical Class	Log2FC (avg) (T6 vs. T0)	Log2FC (avg) (T12 vs. T0)	Most Discriminant Compounds (OPLS-DA)
Amino acids, peptides, and analogues	−0.61	0.40	Val-Pro-Pro (VIP score = 1.89)
Benzenoids	0.19	−1.44	Cresol (VIP score = 1.52)
Carbohydrates and carbohydrate conjugates	0.49	1.39	N-Acetylmannosamine (VIP score = 1.65)
Fatty acyls	1.21	2.31	10Z-Pentadecenoic acid (VIP score = 1.67)
Polyphenols	−0.51	−0.94	trans-Cinnamic acid (VIP score = 1.67)
Purines, Pyridines and Pyrimidines	−0.03	−0.52	Nicotinic acid (VIP score = 1.79)
Steroids and steroid derivatives	−1.56	−0.81	1b-Hydroxycholic acid (VIP score = 1.34)
Diacylglycerols	2.04	4.42	DG(18:3(6Z,9Z,12Z)/16:0/0:0)[iso2] (VIP score = 1.27)
Glycerophosphocolines	−1.69	−2.31	Glycerophosphocholine (VIP score = 1.64)
Triradylglycerols	4.26	4.30	TG(13:0/18:3(9Z,12Z,15Z)/21:0)[iso6] (VIP score = 1.33)
Other compounds	−2.25	−2.83	Mevalonolactone (VIP score = 1.57)

## Supplementary Materials

The following are available online at https://www.mdpi.com/article/10.3390/foods10112722/s1, Table S1: Metabolomic dataset resulting from the UHPLC-QTOF-MS analysis together with the cross-validation parameters and permutation plots of each supervised OPLS-DA method.
